# High-Power Short-Duration Posterior Wall Isolation in Addition to Pulmonary Vein Isolation in Persistent Atrial Fibrillation Ablation Using the New TactiFlex™ Ablation Catheter

**DOI:** 10.3390/jcdd11090294

**Published:** 2024-09-20

**Authors:** Sergio Conti, Francesco Sabatino, Giulia Randazzo, Giuliano Ferrara, Antonio Cascino, Giuseppe Sgarito

**Affiliations:** Department of Electrophysiology, ARNAS Civico-Di Cristina-Benfratelli, 90127 Palermo, Italygiuseppe.sgarito@arnascivico.it (G.S.)

**Keywords:** persistent atrial fibrillation, atrial fibrillation ablation, pulmonary vein isolation, posterior wall isolation, high-power short-duration

## Abstract

Background: The TactiFlex™ ablation catheter, Sensor Enabled™ (Abbott, Minneapolis, MN, USA), is an open-irrigation radiofrequency (RF) ablation catheter with flexible tip technology. This catheter delivers high-power short-duration (HPSD) RF ablations and has been adopted for atrial fibrillation (AF) ablation. HPSD is well-established not only in pulmonary vein isolation (PVI) but also when targeting extra-pulmonary vein (PV) targets. This study aims to determine the safety, effectiveness, and acute outcomes of PVI plus posterior wall isolation (PWI) in patients with persistent atrial fibrillation (Pe-AF) using HPSD and the TactiFlex™ ablation catheter. Methods: Consecutive patients who underwent the ablation of Pe-AF in our centre between February 2023 and February 2024 were prospectively enrolled in the study. All patients underwent PVI plus PWI using TactiFlex™ and the HPSD strategy. The RF parameters were 50 W on all the PV segments and the roof, and within the posterior wall (PW). Left atrial mapping was performed with the EnSite X mapping system and the high-density multipolar Advisor HD Grid, Sensor Enabled™ mapping catheter. We compared the procedural data using HPSD with TactiFlex™ (*n* = 52) vs. a historical cohort of patients who underwent PVI plus PWI using HPSD settings and the TactiCath ablation catheter (*n* = 84). Results: Fifty-two consecutive patients were included in the study. PVI and PWI were achieved in all patients in the TactiFlex™ group. First-pass PVI was achieved in 97.9% of PVs (*n* = 195/199). PWI was obtained in all cases by delivering extensive RF lesions within the PW. There were no significant differences compared to the TactiCath group: first-pass PVI was achieved in 96.3% of PVs (*n* = 319/331). Adenosine administration revealed PV reconnection in 5.7% of patients, and two reconnections of the PW were documented. Procedure and RF time were significantly shorter in the TactiFlex™ group compared to the TactiCath group, 73.1 ± 12.6 vs. 98.5 ± 16.3 min, and 11.3 ± 1.5 vs. 23.5 ± 3.6 min, respectively, *p* < 0.001. The fluoroscopy time was comparable between both groups. No intraprocedural and periprocedural complications related to the ablation catheter were observed. Patients had an implantable loop recorder before discharge. At the 6-month follow-up, 76.8% of patients remained free from atrial arrhythmia, with no significant differences between groups. Conclusions: HPSD PVI plus PWI using the TactiFlex™ ablation catheter is effective and safe. Compared to a control group, the use of TactiFlex™ to perform HPSD PVI plus PWI is associated with a similar effectiveness but with a significantly shorter procedural and RF time.

## 1. Introduction

The cornerstone of any catheter ablation of atrial fibrillation (AF) is to achieve durable pulmonary vein isolation (PVI). Over the last years, the guidelines recommend PVI for the treatment of paroxysmal and persistent atrial fibrillation (Pe-AF) ablation [1,2,3]. However, in the setting of Pe-AF, there is still significant debate on which strategy should be adopted [4,5]. The common ground of all strategies is to achieve a complete and durable ablation. A durable lesion set is a prerequisite to prevent future arrhythmia recurrences due to partially ablated tissue. Extra-pulmonary vein triggers, including the posterior wall (PW), are among the targets recognized to be addressed in Pe-AF to improve ablation outcomes. Several reasons justify the interest in performing posterior wall isolation (PWI) when ablating Pe-AF. The first reason is embryological. Indeed, the posterior wall (PW) and pulmonary veins (PVs) are strictly related and share the same origin [6,7]. The second reason is related to the intrinsic electrophysiologic characteristics of the PW atrial myocytes [8,9]. The third reason is based on the observation that PW is prone to atrial remodeling, including fibrotic progression and lymphomononuclear infiltration [10,11].

Nevertheless, the literature data on PWI are controverting. The feasibility, safety, and long-term effectiveness of PWI are still debatable. It is essential that we recognize that most data derive from non-homogeneous strategies to achieve the PWI, mixed patient cohorts, single-center studies, or studies with a small sample size. Finally, mostly, delivering durable lesions on the PW is challenging. The regular confirmation of PWI durability before starting the follow-up is essential, demonstrating that PWI during the index procedure is crucial [12]. Several ablation technologies have recently been developed to improve lesion delivery and durability. Among these, the development of contact force (CF)-sensing ablation catheters with the implementation of multiparametric indices, such as the Ablation Index (AI) or Lesion Size Index (LSI), have been associated with enhanced procedural safety and efficacy [13,14]. Recently, the use of high-power (≥50 W) short-duration (HPSD) radiofrequency (RF) ablation has been demonstrated to be safe and effective and to reduce the procedure duration significantly [15,16,17,18]. One of the last innovations in the field of RF is the introduction of a new open-irrigation RF ablation catheter with flexible tip technology. The TactiFlex™ Ablation Catheter, Sensor Enabled™ (Abbott Technologies, Minneapolis, MN, USA) is developed upon the TactiCath™ Ablation Catheter, Sensor Enabled™ platform, with CF feedback provided via fiber optic and white light interferometry and a magnetic sensor for localization in 3D space. The flexible tip design has been shown in preclinical work to enhance tip-tissue stability. Moreover, the ability to flex and direct irrigation flow to the tip-tissue interface enhances cooling and promotes a higher RF power delivery for more effective lesion creation while minimizing the risk of overheating [19].

This study aimed to determine the safety, effectiveness, and acute outcomes of PVI plus PWI in patients with Pe-AF using HPSD and the TactiFlex™ SE ablation catheter.

## 2. Methods

### 2.1. Patient Population

Between February 2023 and February 2024, we prospectively recruited consecutive patients with Pe-AF who underwent PVI plus PWI using the HPSD protocol. Pe-AF was defined as a continuous AF episode lasting longer than 7 days but <1 year [1]. According to the guidelines, all patients have been previously evaluated and had had an indication to perform catheter ablation. Patients’ clinical characteristics were recorded from the hospital’s medical records. The local institutional review board approved the study protocol, and the study complied with the Declaration of Helsinki. All patients gave written informed consent before the procedure.

### 2.2. Ablation Procedure

**Figure 1:**
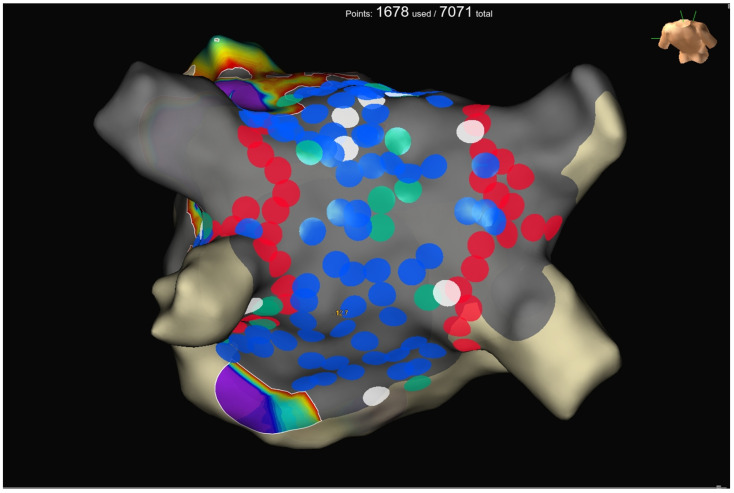
Posterior view of a left atrial substrate map after ablation. Standard lesion set to perform PVI plus PWI. Red dots are for PVI, and blue/white/green dots are for PWI. Blue dots for lesions of 10 s, green dots for lesions between 5 and 10 s, and white dots for lesions lasting less than 5 s. At the end of the procedure, complete isolation of the posterior wall and PVs is visible.

A pre-procedural transesophageal echocardiography was performed to exclude left atrial and left atrial appendage thrombosis. Antiarrhythmic drugs (AADs) were discontinued at least three half-lives before the ablation for class I, and four weeks before for amiodarone. All procedures were performed as previously described [20]. Briefly, an uninterrupted anticoagulation strategy was adopted in all cases. Intra-procedural intravenous heparin administration was given with an initial bolus of 50–100 IU/kg, followed by a continuous infusion (1000 IU/h). The activated clotting time was maintained at ≥300 s and checked every 20 min during the procedures. A 6F deflectable decapolar catheter was inserted through the right femoral vein and advanced into the coronary sinus. Transseptal access was obtained twice using a BRK XS needle and two non-deflectable sheaths (SL1 8.5F, Abbott Medical, Abbott Park, IL, USA). LA geometry and high-density bipolar LA voltage (>2000 points) were performed using the EnSite X mapping system and the Advisor HD Grid SE. A baseline bipolar LA voltage map was created in sinus rhythm before ablation. PVI was performed using the TactiFlex™ SE ablation catheter in a point-by-point fashion. According to the manufacturer, RF was delivered for 10–11 s at 50 W. RF delivery was initiated when a stable CF in the range between 5 and 20 g was reached, apart from ablation sites close to the esophagus, where our target CF was lowered to 5–8 g. PWI was achieved by creating an anterior roof line connecting the antrum of the superior PVs and a caudal line at the floor level of the LA. In addition, as part of our standard protocol for PWI, additional RF lesions across the entire PW were delivered (Figure 1). All procedures were performed using an esophageal probe to monitor the endoluminal temperature (Esotherm Plus, Fiab, Florence, Italy). RF was stopped if the endoluminal esophageal temperature reached 38 °C, which is considered the cut-off limit. The acute endpoint was to achieve complete PVI and PWI, confirmed by the Advisor HD Grid, SE positioned in each PV and by differential pacing maneuvers. After PVI, we observed a waiting time of 20 min for the last ablation. PVI was rechecked with the Grid to assess for spontaneous PV reconnection. If PV reconnection was not documented, intravenous adenosine was given to unmask dormant conduction. CF data were recorded for PVI and PWI. RF, fluoroscopy, procedural times, and incidence of procedural and periprocedural complications (vascular complications, cardiac tamponade, thromboembolism, atrio-esophageal fistulas, phrenic nerve palsy, PVs stenosis, etc.) were also collected. After the procedure, all patients received an implantable loop recorder (Reveal LinQ Medtronic, Minneapolis, MN, USA, or Jot Dx, Abbott Medical, Abbott Park, IL, USA). Before discharge, a transthoracic echocardiography was performed to exclude pericardial effusion.

### 2.3. Patient Follow-Up

All patients recruited in the study completed a visit in the outpatient clinic at 1, 3, 6, and 12 months. At each visit, a standard 12-lead ECG was recorded. Oral anticoagulants were discontinued according to the CHA_2_DS_2_-VASc eight weeks after ablation. AADs were withdrawn at three months or continued at the physician’s discretion. In addition, after the 90-day blanking period, data recorded from the ILR were collected remotely and on-site to evaluate the occurrence of atrial tachycardia (AT), atrial flutter (AFL), and AF episodes. Each follow-up focused on the evaluation of atrial-arrhythmia-related symptoms and AF burden. Atrial arrhythmia recurrence was defined as any documented episode of atrial tachycardia (AT), atrial flutter (AFL), and AF lasting longer than 30 s. The AF burden was calculated as the percentage of time in AF between each follow-up visit based on manually adjudicated episodes. Any arrhythmia observed within three months after ablation was defined as early AF and not considered an arrhythmia recurrence. Redo was always performed after the 90-day blanking period.

### 2.4. Statistical Analysis

This was a single-center prospective study. All clinical characteristics are reported as descriptive statistics. Continuous variables are expressed as mean ± standard deviation. Categorical variables were reported as percentages. A *p*-value of <0.05 was considered statistically significant. All statistical tests were performed using SPSS (v. 25.0) for Windows 25.0 (SPSS, Chicago, IL, USA).

## 3. Results

A total of 52 patients with symptomatic and drug-refractory Pe-AF were consecutively included in the study. The baseline clinical characteristics of the patient population are reported in Table 1. The procedural characteristics are reported in Table 2. In all cases, PWI using HPSD settings was performed after PVI. First-pass PVI was achieved in 97.9% of PVs (*n* = 195/199). First-pass roofline block was obtained in most patients (*n* = 46, 88.4%), while first-pass block of the bottom line was only achieved in 55.7% (*n* = 29). When comparing the HPSD group using the TactiFlex™ catheter to an HC group of patients in which PWI was performed using HPSD (50 W) with the TactiCath catheter, there were no significant differences: first-pass PVI was achieved in 96.3% of PVs (*n* = 319/331), first-pass roofline block in 88.1%, and bottom-line in 52.6% of patients (*p* = ns). Scattered RF applications—in HPSD—within the PW were delivered to obtain a complete PWI. We observed the electrical reconnection of the PVs in 9.6% of patients (*n* = 5/52) and PW reconnection in 3.8% (*n* = 2/52) of patients after adenosine administration. The duration of the procedure and radiofrequency application was significantly shorter in the HPSD group using the TactiFlex™ catheter compared with the historical control group using the TactiCath catheter, 73.1 ± 12.6 vs. 98.5 ± 16.3 min, and 11.3 ± 1.5 vs. 23.5 ± 3.6 min, respectively, *p* < 0.001 (Table 3). The fluoroscopy time was comparable between both groups. No procedural complications related to HPSD settings were observed. One patient had a vascular complication but did not require surgery. The mean length of hospital stay was 1 ± 1.5 days. The mean follow-up is 10.2 ± 3.8 months (median 10.5 months). At the 6-month follow-up, 76.8% of patients remained free from atrial arrhythmia. There were no significant differences compared to the historical control group (76.8% vs. 74.2%, *p* = ns). At the 6-month follow-up, 43.9% of patients were on AADs. The post-procedural AF burden was significantly decreased from 91% to 19% (*p* < 0.0001).

## 4. Discussion

This study assessed the feasibility and safety of the TactiFlex™ SE ablation catheter to perform PVI plus PWI to treat patients with Pe-AF. According to our protocol to perform PWI, we included only patients with Pe-AF in which an extensive PWI has been performed with HPSD. In addition, our patients were strictly followed up with implantable continuous monitors. Compared with a group of Pe-AF patients treated with HPSD (50 W) but with the TactiCath SE ablation catheter, we found an even shorter procedure and RF time but no differences in outcomes or complications. A significant reduction in procedural time may be explained for several reasons. First, the main driver is the reduction in RF time. Second, we noticed that, during PWI, the esophageal temperature increased more frequently in the TactiCath group. Subsequently, a longer time waiting for the temperature to cool down has to be considered. Finally, the TactiFlex™ SE is more stable and maneuverable than the TactiCath SE ablation catheter, and the addition of the direction vector helps to understand which curve and direction the catheter is pointing to.

The biophysics of RF ablation implies the creation of thermal lesions in cardiac tissue. The objective is to increase the tissue temperature to roughly 50 °C, inducing myocardial necrosis. This process is in two sequential stages: the resistive phase and the conductive heating phase. HPSD ablation creates lesions wider and shallower than standard settings, most likely due to the increased resistive heating component [21,22]. Previous studies evaluating catheters without CF-sensing capabilities showed that the high-power RF ablation setting (50 W) resulted in a better long-term freedom-from-AF with shorter fluoroscopy and procedural times without increasing the complication rates when compared to low-power (35 W) ablations [23,24,25]. 

PWI is feasible as an adjunct strategy to PVI for the catheter ablation of Pe-AF [26,27,28,29,30]. A recent meta-analysis, including randomized clinical trials, confirmed these results and demonstrated the incremental benefit of PWI [31]. However, the strategy adopted to perform PWI remains unstandardized and is technically challenging. For these reasons, PWI remains a debatable and controversial point. An effective, safe, and durable PWI is technically laborious because of the complex anatomical structure of the atrial musculature and the close relationship with extracardiac structures. Although PWI performed by creating lines of block seems to be the most common strategy adopted, it may have some drawbacks. Indeed, even if a conduction block along the lines is achieved, the occurrence of gaps over time cannot be ruled out, and thus dormant conduction may take place during the follow-up. Tamborero et al. reported that PWI achieved with linear lesions does not improve the clinical outcome of PVI [32]. In their paper, nearly 70% of patients had a reconnection of the roof line or recurrence of electrical activity within the PW that led to AF and AFL relapses. Similar findings have been reported by Sayuri et al., showing a reconnection of PW in 65% of patients after the second procedure [33]. Disappointing results have been reported in the CAPLA randomized clinical trial, which assessed the role of empirical PWI in patients with Pe-AF [34]. The trial did not show additional advantages in the group of patients randomized to PWI, again raising doubts about this approach. Nevertheless, among the criticisms raised to this study, in particular, the technique adopted to obtain PWI was criticized. Due to the different left atrial wall thicknesses and complex orientations of the myocardial fibers within the PW, creating a standard linear lesion set may not be enough. Indeed, previous studies have shown a high reconnection rate when PWI is carried out using a “box” lesion set and low power (20–35 W). Moreover, the authors created the box lesion by placing a single roof and floor line connecting bilateral PV-encircling lesions’ superior and inferior ends. Extra-PV triggers may originate from the whole area of the PW, including the bottom of the LA [35]. In the PRECEPT study, a different approach to achieving PWI was evaluated, and a higher single-procedure success rate was reported in Pe-AF (80.4% at 15 months). [36]. We reported a slightly lower success rate (71.4% at 12 months) using a similar approach to isolate the PW with the previous generation of the ablation catheter delivering HPSD [37]. Finally, the role of PW needs to be reconsidered even if the CAPLA study demonstrated that PWI would not enhance the ablation outcome in Pe-AF because its isolation appears beneficial if the PW is the AF driver [38]. 

The widespread use of pulsed-field ablation (PFA) in clinical practice may change the future of extra-PV ablation. Although the most widely used device has been developed for PVI, some data are also available regarding the use of the pentaspline multielectrode PFA ablation catheter, showing its safety and efficacy. Ollitrault et al. reported an overall good safety profile with no complications in a prospective multicenter study in which superior vena cava isolation was performed with the pentaspline multielectrode PFA ablation catheter [39]. Similarly, Kueffer et al. recently reported their experience using the pentaspline PFA catheter for PWI. The device, in flower shape, is well-suited for PWI without needing a touch-up with thermal ablation. However, the authors pointed out a crucial point of this procedure. An optimal lesion overlap is required to cover the PW area completely, and good contact with the PW must be ensured. Today, the absence of an integrated and accurate visualization of the pentaspline PFA catheter on a 3D mapping system may be a limitation of this approach [40].

## 5. Limitations of the Study

This study has some limitations related to its design. This was a prospective but non-randomized and single-center study. Although several experiences and this study confirm the feasibility of HPSD ablation for treating Pe-AF, our results may not be reproducible. In addition, the number of patients enrolled is quite limited. We compared the HPSD treatment group with an HC group, and, for this reason, we cannot definitively conclude on the role of HPSD in performing PWI on top of PVI. Although the sample size was inadequate for evaluating the overall safety, we did not observe any procedural-related complications. Larger and randomized data with a longer follow-up duration are needed to validate these data. Finally, roughly half of the patients were on continuous AADs even after the blanking period, limiting us from accurately assessing the correlation between PWI and the outcome.

## 6. Conclusions

The edoption of HPSD PVI plus PWI using the TactiFlex™ ablation catheter seems effective and safe. Compared to a control group, the use of TactiFlex™ to perform HPSD PVI plus PWI is associated with a similar effectiveness but with a significantly shorter procedural and RF time. Our findings need to be validated in larger, multicenter, and randomized studies.

## Figures and Tables

**Table 1:** Baseline clinical characteristics.

	TactiFlex™ Group (*n* = 52)	TactiCath Historical Control Group (*n* = 84)
Male, *n* (%)	32 (61.5)	49 (58.3)
Age, mean ± SD	62.4 ± 13.5	63.1 ± 11.8
Duration of AF, months (mean ± SD)	10.7 ± 3.6	10.6 ± 3.2
Hypertension, *n* (%)	30 (57.6)	46 (54.7)
Diabetes, *n* (%)	6 (11.5)	9 (10.7)
Renal failure, *n* (%)	4 (7.6)	6 (7.1)
Dyslipidemia, *n* (%)	13 (25)	20 (23.8)
OSAS, *n* (%)	9 (17.3)	13 (15.4)
COPD, *n* (%)	5 (9.6)	7 (8.3)
Active smoker, *n* (%)	7 (13.4)	12 (14.2)
BMI, mean ± SD	28.7 ± 4.8	29.1 ± 4.6
CHA_2_DS_2_-VASc, mean ± SD	2.8 ± 0.6	2.9 ± 0.8
HASBLEED score, mean ± SD	1.4 ± 0.7	1.6 ± 0.8
LA diameter, mm (mean ± SD)	48.5 ± 12.3	48.2 ± 13.2
LA area, cm^2^ (mean ± SD)	33.1 ± 9.3	32.9 ± 8.9
LA volume, mL (mean ± SD)	67.2 ± 15.8	66.8 ± 15.3
Indexed LA volume, mL/m^2^ (mean ± SD)	33.7 ± 8.1	33.8 ± 7.1
LVEF, mean ± SD	55.7 ± 11.3	56.3 ± 11.2
Tachycardiomyopathy, *n* (%)	6 (11.5)	8 (9.5)
EHRA class IIa, *n* (%)	9 (17.3)	22 (26.1)
EHRA class IIb, *n* (%)	28 (53.8)	34 (40.4)
EHRA class III, *n* (%)	15 (28.8)	28 (33.3)
ICM, *n* (%)	9 (17.3)	14 (16.6)
DCM, *n* (%)	4 (7.6)	6 (7.1)
HCM, *n* (%)	3 (5.7)	4 (4.7)
Baseline therapy		
- Beta-blockers, *n* (%)	14 (40)	18 (39.1)
- Class IC, *n* (%)	3 (8.5)	5 (10.8)
- Amiodarone, *n* (%)	26 (74.3)	34 (73.9)
- Sotalol, *n* (%)	5 (14.2)	7 (15.2)

AF = atrial fibrillation; OSAS = obstructive sleep apnea syndrome; COPD = chronic obstructive pulmonary disease; BMI = body mass index; LA = left atrium; LVEF = left ventricular ejection fraction; ICM = ischemic cardiomyopathy; HCM = hypertrophic cardiomyopathy; DCM = dilated cardiomyopathy.

**Table 2:** Procedural characteristics.

	TactiFlex Group (*n* = 52)
Pre-procedural TEE, *n* (%)	52 (100)
Procedural duration, min (mean ± SD)	61.3 ± 10.3
Total RF time, min (mean ± SD)	11.2 ± 1.5
ICE, *n* (%)	11 (21.1)
US-guided femoral puncture, *n* (%)	10 (19.2)
Double transeptal puncture, *n* (%)	48 (92.3)
**PVI**	
LPV common ostia, *n* (%)	5 (9.6)
RPV common ostia, *n* (%)	0
Intermediate / accessory PVs, *n* (%)	2 (3.8)
PVI, *n* (%)	52 (100)
WACA, *n* (%)	7 (13.4)
WACA + carina, *n* (%)	45 (86.6)
PVs isolated at first pass during PVI, *n* of PVs (%)	195/199 (97.9)
CF on anterior LPVs, (mean ± SD)	13.1 ± 4.6
CF on posterior LPVs, (mean ± SD)	11.3 ± 3.8
CF on anterior RPVs, (mean ± SD)	15.1 ± 2.9
CF on posterior RPVs, (mean ± SD)	10.6 ± 2.3
Adenosine, *n* (%)	52 (100)
PV acute reconnection, *n* (%)	3 (5.7)
**PWI**	
PWI, *n* (%)	52 (100)
RF time on PW, (mean ± SD)	3.4 ± 1.5
First-pass roofline block, *n* (%)	46 (88.4)
First-pass bottom line block, *n* (%)	29 (55.7)
First-pass PWI, *n* (%)	27 (51.9)
CF on PW, g (mean ± SD)	11.4 ± 2.3
Adenosine, *n* (%)	52 (100)
PW acute reconnection, *n* (%)	2 (3.8)

TEE = transesophageal echocardiography; ICE = intracardiac echocardiography; US = ultrasound; LPV = left pulmonary vein; RPV = right pulmonary vein; PVI = pulmonary vein isolation; WACA = wide antral circumferential ablation; PW = posterior wall; CF = contact force.

**Table 3:** TactiFlex vs. TactiCath (control group).

	TactiFlex (*n* = 52)	TactiCath (*n* = 84)	*p*
Procedural duration, min (mean ± SD)	73.1 ± 12.6	98.5 ± 16.3	<0.001
Total RF time, min (mean ± SD)	11.3 ± 1.5	23.5 ± 3.6	<0.001
RF time on PVs, min (mean ± SD)	8.2 ± 1.6	17.3 ± 3.1	<0.001
RF time on PW, min (mean ± SD)	3.3 ± 1.2	6.2 ± 1.4	<0.001
Fluoroscopy time, min (mean ± SD)	4.2 ± 2.7	4.3 ± 2.8	ns
Double transeptal puncture, *n* (%)	48 (92.3)	72 (85.7)	ns
PVs isolated at first pass during PVI, % (*n* of PVs)	97.9 (195/199)	96.3 (319/331)	ns
PV acute reconnection, *n* (%)	2 (3.8)	7 (8.3)	ns
First-pass roofline block, *n* (%)	46 (88.4)	74 (88.1)	ns
First-pass bottom line block, *n* (%)	29 (55.7)	44 (52.6)	ns
First-pass PWI, *n* (%)	27 (51.9)	38 (45.2)	<0.05
PW acute reconnection, *n* (%)	2 (3.8)	5 (5.9)	ns

RF = radiofrequency; PVs = pulmonary veins; PW = posterior wall; PVI = pulmonary vein isolation; PWI = posterior wall isolation.

## Data Availability

The data are available upon reasonable request.
